# Fibroblast-like synovial cell production of extra domain A fibronectin associates with inflammation in osteoarthritis

**DOI:** 10.1186/s41927-019-0093-4

**Published:** 2019-12-02

**Authors:** Tue W. Kragstrup, Dong H. Sohn, Christin M. Lepus, Kazuhiro Onuma, Qian Wang, William H. Robinson, Jeremy Sokolove

**Affiliations:** 1 Department of Immunology and Rheumatology, Stanford University, Stanford, CA USA; 2 VA Palo Alto Health Care System, Palo Alto, CA USA; 3 Department of Biomedicine, Aarhus University, Wilhelm Meyers Allé 4, DK-8000 Aarhus C, Denmark; 4 Department of Rheumatology, Aarhus University Hospital, Aarhus, Denmark; 5 Department of Microbiology and Immunology, Pusan National University School of Medicine, Yangsan, Republic of Korea

**Keywords:** Osteoarthritis, Fibronectin, Myofibroblast, Inflammation, Arthritis, Synovitis, Synoviocyte, Drug delivery

## Abstract

**Background:**

The pathophysiology of osteoarthritis (OA) involves wear and tear, and a state of low-grade inflammation. Tissue repair responses include transforming growth factor beta (TGFβ)-induced myofibroblast production of extracellular matrix. Fibronectins are an essential part of the extracellular matrix, and injection of fibronectin fragments into rabbit joints is a previously established animal model of OA. Fibronectin containing the ED-A domain is currently being used as drug delivery target in the development of anti-inflammatory drugs (e.g. Dekavil).

**Methods:**

In this study, samples of synovial membrane were obtained from patients with knee OA undergoing joint replacement surgery. Immunostaining for ED-A fibronectin and the myofibroblast marker alpha smooth muscle actin (αSMA) was performed on fibroblast-like synovial cells (FLS) and synovial membranes. RAW 264.7 macrophages were incubated with recombinant ED-A fibronectin.

**Results:**

The staining of ED-A fibronectin in OA FLS was increased by TGFβ but not by TNFα, lipopolysaccharide, or IL-6 (*n* = 3). ED-A fibronectin co-stained with the myofibroblast marker αSMA in both the OA FLS (*n* = 3) and in the OA synovial membranes (*n* = 8). ED-A fibronectin staining was associated with both number of lining layer cells (rho = 0.85 and *p* = 0.011) and sublining cells (rho = 0.88 and *p* = 0.007) in the OA synovium (*n* = 8), and co-distributed with TNFα (*n* = 5). Recombinant ED-A fibronectin increased the production of TNFα by RAW 264.7 macrophages (*n* = 3).

**Conclusions:**

The disease process in OA shares features with the chronic wound healing response. Our findings support utilizing ED-A fibronectin for drug delivery or therapeutic targeting to reduce pro-inflammatory responses in OA.

## Background

Osteoarthritis (OA) is a very common disease affecting approximately 60% of the population by age 65 years with no current therapeutics approved for preventing disease progression. The disease is often restricted to a few joints and with very low systemic inflammatory burden. Due to the slow progression of the disease there is a need for treatments with a very mild adverse effect profile. The extracellular matrix molecule fibronectin containing the ED-A domain is currently being used as drug delivery target in the development of anti-inflammatory drugs (e.g. Dekavil) [1, 2]. Here, we show that the expression of fibronectin containing ED-A is closely linked to the pathogenic processes in OA. This could translate into the development of drugs suited for the treatment of OA.

The pathogenesis of OA involves wear and tear and a state of low-grade inflammation, which has generated the hypothesis that OA could be a chronic wound [3–5]. Thus, wear and tear leads to tissue degradation followed by tissue repair responses including transforming growth factor beta (TGFβ)-induced myofibroblast production of extracellular matrix [6]. Extracellular matrix molecules and breakdown products can then function as danger associated molecular patterns leading to activation of the immune system through activation of Toll-like receptors (TLRs) on macrophages [7].

Fibronectins (fibronectins) are an essential part of the extracellular matrix. The active fibronectins consist of several isoforms and peptide fragments with individual functions [8]. Fibronectin produced by fibroblasts is thus a result of both alternative splicing potentially incorporating extra domain A (ED-A) or ED-B and proteolytic cleavage by enzymes such as plasmin and thermolysin [9].

The association between fibronectin and OA is already established [10, 11]. Injection of fibronectin fragments in rabbit joints lead to many characteristics of OA including cartilage degradation and bony spur formation and is now an established animal model of OA [12]. Fibronectin fragments are present in OA synovial fluid and have been shown to induce pro-inflammatory cytokines and matrix metalloproteinases [13, 14]. However, the mechanisms coupling fibronectin fragments and OA are not fully understood. The fibronectin fragments containing the ED-A domain are of particular interest because they show properties with possible implications for both immune activation and joint damage in OA [15]. These fragments have thus recently been shown to function as TLR-4 agonists [16], and synovial fluid levels correlate with establishment of disease and radiographic progression in rheumatoid arthritis [17, 18]. Also, ED-A fibronectin is produced by myofibroblasts and are essential for normal wound healing fitting the theory of OA as a chronic wound [19–21].

In this study, we hypothesize that ED-A fibronectin fragments represent a factor transducing repair mechanisms with inflammatory signals in OA. The aim was to describe the localization and production of ED-A FN in the OA synovium and to explore associations between ED-A FN and inflammation.

## Methods

### Osteoarthritis patients

Samples were obtained from 12 patients with knee OA undergoing joint replacement surgery. Patients were diagnosed with knee OA of Kellgren-Lawrence score 2 to 4 according to the 1985 criteria of the American Rheumatism Association [22, 23]. The number of patients used in each experiment is stated in the figure legends.

### Ethics, consent and permissions

Samples were obtained under protocols approved by the Stanford University Institutional Review Board and with the patients’ informed consent.

### Isolation of synovial tissue and synovial FLSs

Synovial tissue samples for immunofluorescence were snap frozen in Tissue-Tek. FLSs for immunofluorescence were isolated after enzymatic digestion of the tissue. Synovium was minced with sterile scissors digested with collagenase grade II (Clostridium histolyticum) in DMEM and antibiotics supplemented with 5% fetal bovine serum (FBS). The resulting cell suspension was pipetted through a 70 μm mesh and centrifuged at 250 g for 10 min. The cells were washed and incubated at 37 °C and 5% CO_2_. Cells were passaged using trypsin and used at passage 5.

### Immunoflourescence of osteoarthritis FLSs

Immunoflourescence of FLSs were done as previously described [24]. Sterile glass slides were placed in 24-well cell culture plates. FLSs were then seeded at a concentration of 5.0 × 10^4^ cells/mL in DMEM and antibiotics supplemented with 5% FBS and incubated for 24 h at 37 °C. The cells were either untreated or stimulated with TGFβ1 at 5 ng/ml, TNFα (PeproTech) at 10 ng/mL, lipopolysaccharide (LPS) (Sigma) at 100 ng/ml, or IL-6 (PeproTech) at 10 ng/ml as done previously [25–27]. Cells were fixed with 4% paraformaldehyde and then incubated for 30 min at room temperature with PBS with 0.05% Tween20, 1% bovine serum albumin, 5% normal goat serum, and 0.03% NaN_3_. Cells were stained with rabbit polyclonal anti-EDA fibronectin (kind gift from Digna Bioscience, Madrid, Spain) and mouse IgG2a anti-αSMA (clone 1A4, R&D Systems). Rabbit polyclonal isotype and mouse IgG2a isotype were used as negative controls. Goat anti-rabbit alexa 488 and goat anti-mouse IgG2a alexa 555 were used as secondary antibodies (both Thermo Fischer). Wells were washed twice, and glass slides were mounted with Prolong Gold Antifade with DAPI (Thermo Fischer). All micrographs were collected by using a Zeiss LSM-710 confocal microscope. ED-A fibronectin positive cells and total cells were counted for each culture condition in two fields of view counting at least 150 cells for each experimental condition.

### Immunoflourescence of osteoarthritis synovial membranes

Synovial tissue samples for immunofluorescence were snap frozen in Tissue-Tek and cut in sections of 10 μm using a cryostat and sections were fixed with 4% paraformaldehyde and then incubated for 30 min at room temperature with PBS with 0.05% Tween20, 1% bovine serum albumin, 5% normal goat serum, and 0.03% NaN3. Synovial tissue sections were stained with rabbit polyclonal anti-EDA fibronectin (kind gift from Digna Bioscience, Madrid, Spain), mouse IgG2a anti-αSMA (clone 1A4, R&D Systems), rabbit polyclonal anti-αSMA (Abcam), mouse IgG2a anti-CD45 (clone F10–89-4, Abcam), mouse IgG1 anti-CD31 (clone JC70A, Dako), and rabbit polyclonal anti-TNFα (Abcam). Rabbit polyclonal isotype, mouse IgG2a isotype, mouse IgG1 isotype, and mouse IgG2b isotype were used as negative controls. Goat anti-rabbit alexa 488, goat anti-mouse IgG2a alexa 555, goat anti mouse IgG1 alexa 647, and goat anti-mouse IgG alexa 555 were used as secondary antibodies (all Thermo Fischer). Slides were washed twice, and glass slides were placed in 2 μL of Prolong Gold Antifade with DAPI (Thermo Fischer) and allowed to dry overnight. All micrographs were collected with a Zeiss LSM-710 confocal microscope. Micrographs were analyzed using ImageJ software (NIH). The area with ED-A staining was found and calculated as a percentage of total area. The area with DAPI staining in the sublining layer was found and calculated as a percentage of total area. The lining layer cell thickness was counted.

### Stimulation of RAW 264.7 macrophages

RAW 264.7 macrophages were cultured with recombinant human ED-A fibronectin fragment (kind gift from Digna Bioscience, Madrid, Spain) at 10 μg/ml. For confirmation that endotoxin contamination was not a confounding factor, 1) the cells were cultured in the presence of the LPS blocking polypeptide polymyxin B at 15 μg/ml (Sigma-Aldrich), 2) ED-A fibronectin was pretreated with boiling for 30 min, or 3) ED-A fibronectin was pretreated with proteinase K at 20 μg/ml. For each experiment, an untreated cell culture with the same number of cells in medium without stimulants was used for comparison and a culture stimulated with LPS at a concentration of 100 ng/ml was used as a positive control. Cells were cultured for 24 h at 37 °C in a humidified incubator with 5% CO_2_ without changing of medium. After incubation, supernatants were stored frozen at − 80 °C for later assessment.

### Enzyme linked immunosorbent assay

Supernatants from RAW 264.7 macrophage cultures were analyzed for TNFα concentration by ELISA (PeproTech) according to the manufacturors instructions.

### Statistics

Statistical analyses were performed using GraphPad Prism 6.0 for Mac (GraphPad Software). Data with ED-A FN production by OA FLSs were presented as ratios of ED-A^+^ cells divided by total cells. Ratios were log transformed and analyzed with the paired t-test. Correlations of ED-A FN staining and histological features of the synovial membrane were made using Spearman’s Rho. Data with TNFα production by RAW 264.7 cells after ED-A FN stimulation were presented as ratios of stimulated cells divided by untreated cells. In all tests, the level of significance was a two-sided *P* value of less than 0.05. The statistics used in each experiment is also stated in the figure legends.

## Results

### ED-A fibronectin is produced by OA FLSs in response to TGFβ

**Fig. 1:**
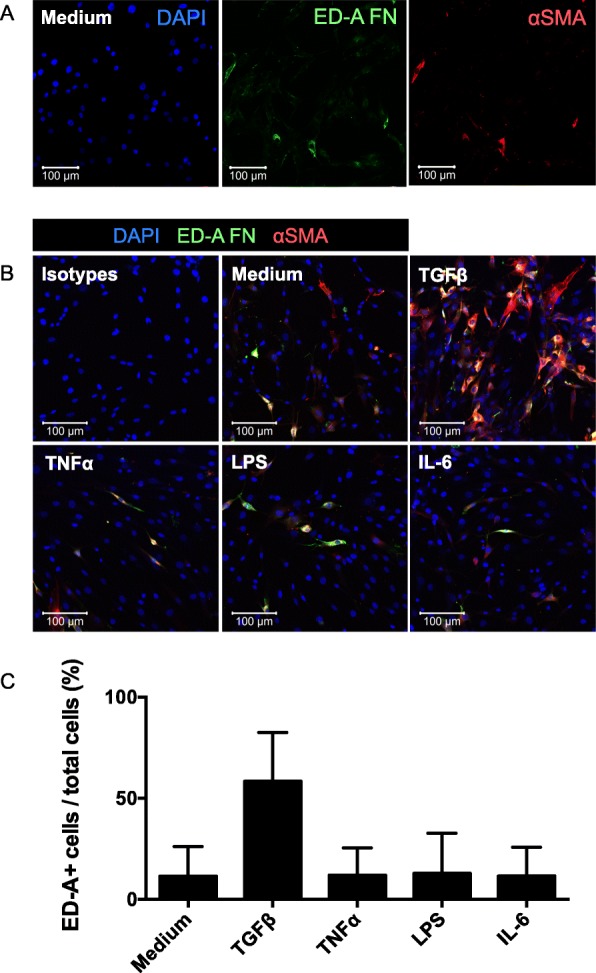
ED-A fibronectin production by OA FLSs. **a** and **b**. Representative confocal microscopy images of αSMA (red) and ED-A fibronectin (green) in OA FLS cultures incubated with medium, TGFβ, TNFα, LPS, or IL-6 (*n* = 3). No staining was seen using isotype control antibodies. **c** ED-A fibronectin was expressed as a ratio of ED-A fibronectin positive cells divided by the total cell count. Data were log transformed and analyzed with the paired t-test. Bars indicate the median and whiskers indicate the IQR. * *P* < 0.05

We first tested whether ED-A fibronectin is produced by OA FLSs in response to TGFβ, TNFα, LPS, and IL-6. OA FLSs were incubated with these stimulators and stained for ED-A fibronectin and the myofibroblast marker αSMA. Spontaneous production of ED-A fibronectin was found in a small number of cells in untreated cultures (Fig. 1a). ED-A fibronectin co-stained with αSMA (Fig. 1a). The number of ED-A FN positive cells divided by total number of cells was increased by TGFβ (*p* = 0.046) (*n* = 3) (Fig. 1b-c). There was no change in the number of ED-A fibronectin positive OA FLSs when using TNFα (*p* = 0.5), LPS (*p* = 0.6), or IL-6 (*p* = 0.9) (Fig. 1b-c). No signal was detected when staining with negative control isotype antibody (Fig. 1b).

### ED-A fibronectin is located to αSMA positive myofibroblasts in OA synovium

**Fig. 2:**
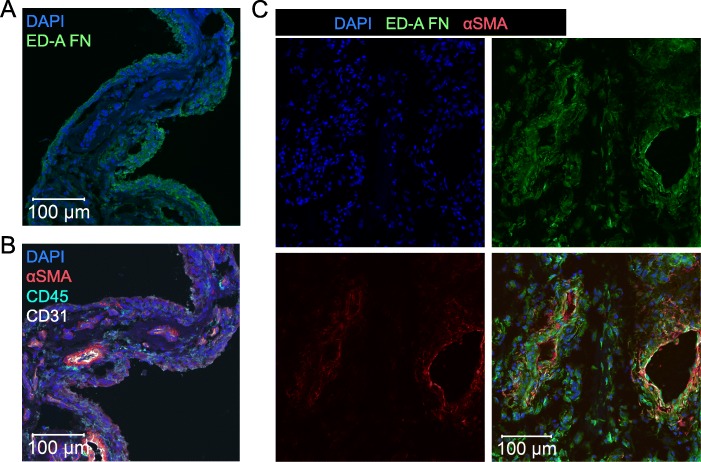
ED-A fibronectin expression in OA synovium. **a** and **b** Representative confocal microscopy images of CD45, CD31, αSMA and ED-A fibronectin in OA synovium (*n* = 8). **c** Representative confocal microscopy images of ED-A fibronectin co-localization with αSMA (*n* = 3)

We then tested whether ED-A fibronectin also co-distributed with αSMA in the OA synovial membrane. ED-A fibronectin was found in the synovium from all OA patients (*n* = 8) (Fig. 2a). The ED-A fibronectin staining was most intense in lining layer cells while αSMA staining was most intense in cells surrounding CD31 positive blood vessels (Fig. 2b). However, most ED-A fibronectin positive cells were also to some extent αSMA positive in all the stained synovial membranes (*n* = 3) (Fig. 2c).

### ED-A fibronectin associates with increased number of lining layer cells and sublining cells in OA synovium

**Fig. 3:**
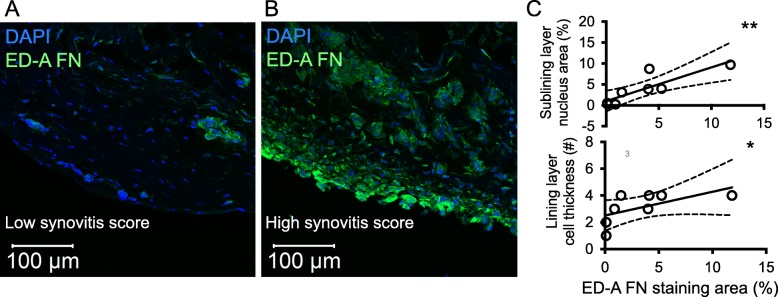
ED-A fibronectin expression and degree of synovitis in OA synovium. **a** and **b** Representative confocal microscopy images of ED-A fibronectin staining and synovitis score (*n* = 8). **c** ED-A fibronectin expression associated with cell infiltration in the sublining layer and cell thickness of the lining layer. Data were analyzed using the Spearman’s Rho. * *P* < 0.05, ** *P* < 0.01

Next, we tested whether ED-A fibronectin expression associates with number of cells in the OA synovium. Therefore, we first analyzed the association between the degree of ED-A fibronectin staining and the number of lining layer cells and sublining cells in OA synovium. The ED-A fibronectin staining associated with both number of lining layer cells (rho = 0.85 and *p* = 0.011) and sublining cells (rho = 0.88 and *p* = 0.007) in OA synovium (*n* = 8) (Fig. 3a-c).

### ED-A fibronectin is located to areas of TNFα staining in OA synovium

**Fig. 4:**
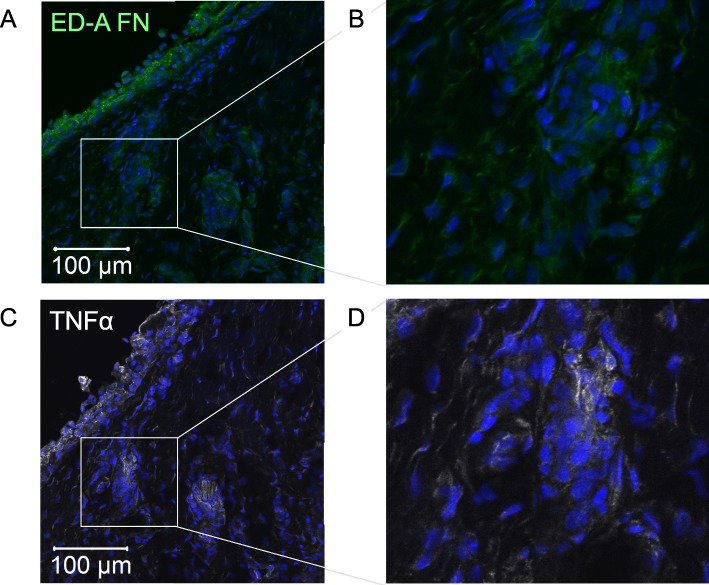
Co-localization of ED-A fibronectin. **a**-**d** Representative confocal microscopy images of localization of ED-A fibronectin and TNFα in OA synovium (*n* = 5). TNFα staining was found in areas with ED-A fibronectin staining. **c** and **d** Close-up of the white boxes on original images

We then analyzed the OA synovial membranes for co-distribution of ED-A fibronectin and TNFα. ED-A fibronectin and TNFα were stained on serial slides. ED-A fibronectin co-distributed with TNFα in all stained synovial membranes (*n* = 5) (Fig. 4a-b). The staining of TNFα was mostly located to cells in close proximity to the ED-A fibronectin positive cells but not specifically to the ED-A fibronectin positive cells.

### Recombinant ED-A fibronectin increases the secretion of TNFα by RAW 264.7 macrophages

**Fig. 5:**
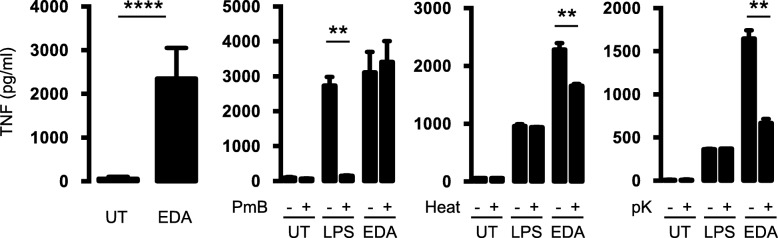
Effect of ED-A fibronectin on TNFα production. **a**-**c** RAW 264.7 cells incubated untreated (UT), with LPS (100 ng/ml), or with recombinant ED-A fibronectin (10 μg/ml). TNFα concentration in supernatants of cultures after 24 h. **a** Effect of ED-A fibronectin (*n* = 9). **b** Cells cultured with or without polymyxin B at 15 μg/ml (PmB) (*n* = 3). **c** ED-A fibronectin with or without pretreatment with boiling for 30 min (Heat) (*n* = 3). **d** ED-A fibronectin with or without pretreatment with proteinase K at 20 μg/ml (pK) (*n* = 3). Data were analyzed with the paired t-test. Bars indicate the median and whiskers indicate the IQR. ** *P* < 0.01

We now tested whether the association between ED-A fibronectin and TNFα in the synovial membrane could be caused by a stimulatory effect of ED-A fibronectin on TNFα production by macrophages. Recombinant ED-A fibronectin increased the production of TNFα from RAW 264.7 cells using a concentration of 10 μg/ml (*p* < 0.0001) (*n* = 9) (Fig. 5). The stimulatory effect of ED-A FN was not reduced with the LPS-blocking polypeptide polymyxin B (*p* = 0.69). In contrast, the stimulatory effect of LPS was significantly decreased with polymyxin B (*p* = 0.0031) (*n* = 3) (Fig. 5). The stimulatory effect of ED-A fibronectin was reduced when treating the ED-A fibronectin with proteinase K (*p* = 0.001) or heat (*p* = 0.0068) (*n* = 3) (Fig. 5). Taken together, this indicates that the effect of ED-A fibronectin was not caused by contamination with LPS.

## Discussion

OA is a common joint disease involving wear and tear and low-grade inflammation. The disease pathogenesis has thus been speculated to resemble a pathogenic wound healing response [3, 4]. Here, we show that ED-A fibronectin could be part of such pathogenic wound healing mechanisms.

First, we studied expression of ED-A fibronectin in OA. We found formation of ED-A fibronectin in αSMA positive myofibroblasts isolated from OA synovial tissue, induction of ED-A fibronectin production by TGFβ, and co-localization of ED-A fibronectin and αSMA in the OA synovium. This is in line with previous findings suggesting that ED-A fibronectin is expressed in TGFβ induced myofibroblast differentiation [8], and that TGFβ is increased in OA synovial fluid [28]. Further, the role of a general wound healing response in OA is supported by findings of increased fibronectin secretion after blunt cartilage trauma in a bovine model of OA [29], increased fibrosis and joint stiffening in OA [30], and by the protective effect of blocking TGFβ in experimental OA in mice [6].

Then, we studied the possible association between ED-A fibronectin and inflammation in OA. We found that ED-A fibronectin is associated with lining layer thickness and sublining cellular density in the OA synovium. This is in line with previous findings of high ED-A fibronectin expression in rheumatoid arthritis and in proliferative regions of OA [31, 32]. The association between ED-A fibronectin expression and cellular density and presence of inflammatory molecules in the OA synovium is of particular interest because there are currently ongoing studies of utilizing ED-A fibronectin as a drug delivery target in rheumatoid arthritis and other diseases [33]. E.g., Dekavil is a drug combining a human F8 antibody specific to the ED-A domain of fibronectin fused to the anti-inflammatory cytokine IL-10 [1, 2]. A recent study, only found small amounts of ED-A fibronectin mRNA in OA synovium [34]. This suggests that the formation of ED-A fibronectin might rather be a result of enzymatic digestion (e.g. by plasmin or thermolysin) or folding than synthesis.

Macrophages are known to be part of the cellular infiltrate in the OA synovium [35]. We therefore studied the effect of recombinant ED-A fibronectin on macrophage production of these pro-inflammatory mediators. The recombinant ED-A fibronectin fragment used in these experiments stimulated the production of TNFα by RAW 264.7 macrophages. The stimulatory effect was not decreased by the LPS-blocking polypeptide polymyxin B and could be diminished by proteinase K treatment or heat. This indicates that the effect of the ED-A FN fragments was not caused by contamination with LPS. This finding was supported by the co-distribution of ED-A FN with TNFα in the OA synovial membrane. Further, our study is in line with previous findings of ED-A fibronectin induced increase of IL-1β production by synovial cells [36]. The stimulatory effects could be due to ED-A fibronectin binding to TLR-4 as previously reported [16].

There are several limitations to this study. Here, only TGFβ, TNFα, LPS, and IL-6 were studied for potential ED-A FN inducing properties in OA FLSs. It is therefore not known whether other cytokines or other factors could induce ED-A FN production in these cells. In the assay using RAW 264.7 macrophages, the positive LPS control stimulation gave very variable results. However, the treatment effect of ED-A FN was seen in all experiments. Therefore, the variations do not seem to have influenced the results. ED-A FN and LPS were both used in only one concentration. Therefore, no comparison can be made from this study. Further, only TNFα was measured as the downstream effect of ED-A FN in macrophages. Other cytokines or chemokines could likely be altered with ED-A FN stimulation. It is also not known from this study how ED-A FN affects human OA macrophages. Further, the present study is limited by the in vitro design. It would be interesting to see the treatment effect of ED-A FN targeting drugs in a mouse model of OA. Finally, this study is limited by a rather small sample size.

There is an unmet therapeutic need preventing OA disease progression. This study supports that ED-A fibronectin could be a target for drug delivery to the inflamed joint or that the formation of ED-A fibronectin or the enzymatic fragmentation of fibronectin could be novel therapeutic targets in OA.

## Conclusions

Taken together, we show that myofibroblasts produce ED-A fibronectin and that ED-A fibronectin stimulates TNFα production by macrophages possibly promoting inflammation in OA.

## Data Availability

Please contact corresponding author TWK for data requests.

## Abbreviations

ED-A: ED-A domain
FBS: Fetal bovine serum
FLS: Fibroblast-like synovial cells
IL: Interleukin
IQR: Interquartile range
LPS: Lipopolysaccharide
OA: Osteoarthritis
PBS: Phosphate buffered saline
TGFβ: Transforming growth factor beta
TLR: Toll-like receptor
TNFα: Tumour necrosis factor alpha
UT: Untreated
αSMA: Alpha smooth muscle actin
